# Initial experience of reduced port surgery using a two-surgeon technique for colorectal cancer

**DOI:** 10.1186/s12893-015-0078-1

**Published:** 2015-07-30

**Authors:** Jo Tashiro, Shigeki Yamaguchi, Toshimasa Ishii, Hiroka Kondo, Kiyoka Hara, Ryuichi Kuwahara

**Affiliations:** Division of Gastroenterological Surgery, Saitama Medical University International Medical Center, Yamane, Hidaka-shi, Saitama 350-1298 Japan

**Keywords:** Reduced port laparoscopic surgery, Colorectal cancer, 2-surgeon technique, Conventional laparoscopic colectomy

## Abstract

**Background:**

With the decreasing number of surgeons on surgical teams, reduced port surgery (RPS) operations have become popular. We herein present our initial experience with RPS, which was successfully performed using a two-surgeon technique. A retrospective analysis was performed to compare the two-surgeon technique with conventional laparoscopic colectomy and evaluate its efficacy.

**Methods:**

A total of 535 patients were eligible among 749 registered patients. Conventional multiport laparoscopic colectomy with three surgeons and RPS using the two-surgeon technique with a surgeon and surgeon’s assistant were performed in 429 and 106 cases, respectively. The patient characteristics, short-term outcomes (including intraoperative and postoperative findings) and pathological results were recorded and analyzed.

**Results:**

The two groups were similar with respect to age, gender, BMI, history of abdominal surgery, depth of tumor invasion and TNM classification. Reconstruction via extracorporeal functional end-to-end anastomosis was performed in a significantly higher number of patients in the two-surgeon technique group (74 %) than in the conventional laparoscopic colectomy group (57 %). Furthermore, the mean operative time in the two-surgeon technique group (117.9 min) was significantly shorter than that observed in the conventional laparoscopic colectomy group (170 min), and the median postoperative hospital stay was significantly shorter in the two-surgeon technique group (6 days) than in the conventional laparoscopic colectomy group (7 days). There were no major postoperative complications. The final TNM stage was similar in both procedures.

**Conclusion:**

RPS using the two-surgeon technique compares favorably with conventional laparoscopic colectomy and is considered to be a safe and successful procedure.

## Background

Laparoscopic colon cancer resection is commonly performed worldwide. This procedure was established in the early 1990’s [1], and major randomized trials have presented evidence that it is associated with better short-term and long-term outcomes than open surgery [2–5]. Conventional laparoscopic colectomy is typically performed by a team of three surgeons, including a surgeon, surgeon’s assistant and laparoscopist. However, there has been a marked decrease in the number of general surgeons in the United States, Europe and Japan. The causes of this shortage include an unfavorable work environment, reimbursement issues, professional liability and, possibly most importantly, a change in the nature of the workforce of individuals who are entering medicine [6, 7]. It is hoped that the two-surgeon technique may be used to relieve the burden on both the patient and surgeon associated with the provision of surgical care in settings with a decreasing number of surgeons. In this way, the two-surgeon technique may be beneficial in clinical practice. The reduction in the number of surgeons has led to the development of reduced port surgery (RPS), which is less invasive and expensive than conventional surgical techniques. Only a surgeon and surgeon’s assistant are required to perform the two-surgeon technique.

Laparoscopic colorectal cancer surgery using a single incision, which is usually made in the umbilical area, has emerged as a surgical option that minimizes scarring and provides better cosmetic results than conventional surgery [8–11]. However, good laparoscopic skills are essential for maintaining the oncologic principles of colorectal cancer surgery within a restricted surgical field during the procedure. RPS may be a bridge between conventional multiport laparoscopic surgery and single-incision laparoscopic colectomy [12–15].

We herein present our initial experience with RPS for colorectal cancer, which was performed successfully using the two-surgeon technique. In addition, we conducted a retrospective analysis to compare the two-surgeon technique with conventional laparoscopic colectomy in order to evaluate the feasibility of this approach.

## Methods

Between June 1, 2012 and August 31, 2014, a total 749 patients with colorectal cancer who underwent laparoscopic surgery at our institution were registered. Two patient groups were retrospectively assigned for the data analysis. Four hundred and nine patients (57 %) received conventional laparoscopic colectomy (five ports, performed by three surgeons) and 106 patients (14 %) received RPS performed by one surgeon and one surgeon’s assistant. Two hundred and fourteen (29 %) patients were excluded from the analysis due to contraindications for RPS using the two-surgeon technique (including synchronous other site cancer resection and rectal cancer). Almost all of these surgeries were performed by three board-certified colorectal surgeons: the first author of this manuscript (J.T.) performed only the two-surgeon technique and the other two surgeons performed conventional procedures. All of the patients participating in the present study provided their informed consent for the RPS operations and publication of their individual clinical details. Access to the patients’ records was also granted by the hospital. This study was approved by the ethics committee of the Saitama Medical University International Medical Center. In-hospital registration of this retrospective study was applied.

The indications for conventional laparoscopic colectomy included colorectal cancer and large adenomas that were not suitable for endoscopic removal. The indications for RPS using the two-surgeon technique were similar, but included the following contraindications: rectal cancer located within 10 cm of the anal verge on imaging and a digital rectal examination; severe and direct invasion of a major organ; bowel obstruction without decompression; morbid obesity; or emergency surgery.

The patients did not receive mechanical bowel preparation. Following the procedure, each patient was cared for on the hospital’s intensive care unit. Walking and fluid intake were started on postoperative day 1, a soft diet was implemented on postoperative day 3 and fluid therapy was postoperatively maintained for four days to prevent dehydration. Patients were recommended for discharge once he or she were ambulant without any major complications.

The patient characteristics, short-term outcomes (including intraoperative and postoperative findings) and pathological results were recorded and analyzed. The present study was associated with some limitations, including the retrospective nature of the clinical observations, lack of patient randomization and dependence on the information available in the patient files. All of the statistical analyses were performed using the SPSS version 21.0 software program (SPSS Inc., Chicago, IL, USA). The *X*^2^ test, Fisher’s exact probability test and Mann–Whitney *U* test were used to compare the two types of surgical procedures. *P* values of < 0.05 were considered to be statistically significant in all analyses.

### RPS with the two-surgeon technique

This procedure was performed under general anesthesia in all patients; we did not administer epidural anesthesia. The surgical wounds were injected with ropivacaine hydrochloride 0.5 % before closure. Postoperative pain was mainly controlled by the patient with an anesthetization pump.

**Figure 1:**
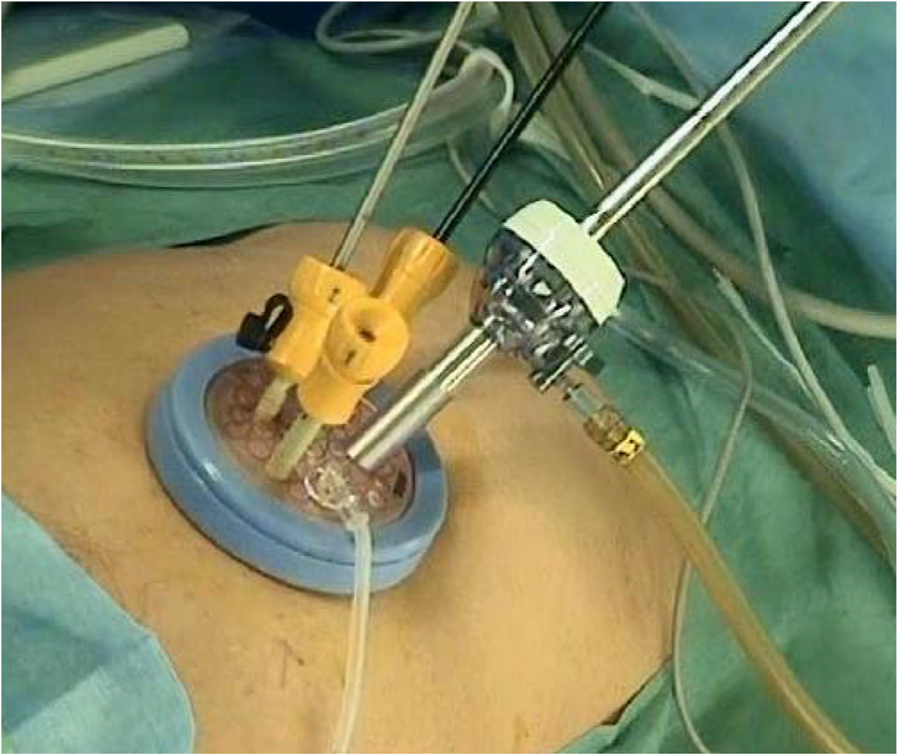
Free Access®

Access was obtained via the umbilical approach using Free Access® (TOP Corporation, Japan; Fig. 1), a newly developed system that incorporates easily controlled settings and simple operability for multi-access or single-port laparoscopic surgery. In this technique, a small wound protector/retractor (Alexis®, Applied Medical, USA) was inserted through a 3–5 cm transumbilical incision, and a Free Access device was attached to an Alexis device with three ports (scope port, 12 mm port and two 5 mm ports).

**Figure 2:**
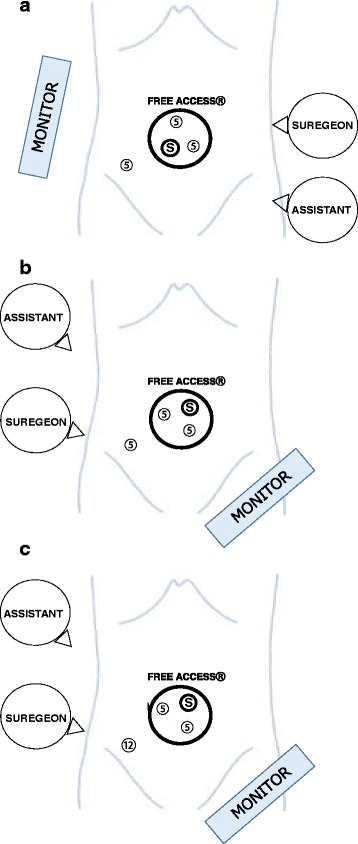
**a** Port placement for right side colon cancer; S, scope; 5, 5 mm port. **b** Port placement for left side colon cancer; S, scope; 5, 5 mm port. **c** Port placement for DST reconstruction; 12, 12 mm port

An additional 3 mm port was placed in the right lower quadrant in patients with right-sided colorectal cancer (Fig. 2a), a 5 mm port was placed on the left side in patients with colon cancer (Fig. 2b) and a 12 mm port requiring rectal transection using a stapling device was placed in patients with sigmoid colon and rectal cancer (Fig. 2c).

This procedure was performed with standard non-articulating instruments and a flexible laparoscope (10 mm; VISERA Pro, Olympus, Japan). The medial-to-lateral approach was used in patients with colorectal cancer. An ultrasonic device (Harmonic ACE®, Ethicon Endo-Surgery, USA) was used for laparoscopic dissection and mobilization.

For partial colectomy, cancer resection and functional end-to-end anastomosis (FEEA) were performed extracorporeally following extraction of the specimen through the Alexis® device (which was placed in the umbilical incision). For cases of rectal cancer, laparoscopic total mesorectal excision and rectal transection after rectal lavage were performed first, prior to lymphadenectomy and mesocolon mobilization. This approach is unique and has been termed the “rectal transection first” approach in our department. The stapling device was applied through the 12 mm port placed in the right lower quadrant. The specimen was then retrieved through the umbilical incision. After inserting an anvil, a circular stapler was used to perform double-stapling technique (DST) anastomosis. We added anastomotic sites to several anchor sutures to release the anastomotic tension. In cases of DST reconstruction, a closed suction drain was placed extending from an additional port site to below the site of anastomosis.

## Results

The characteristics of the patients in the two-surgeon technique group were compared with those of the subjects who underwent conventional laparoscopic colectomy (Table 1). The two groups were similar with respect to age, gender, BMI, history of abdominal surgery, depth of tumor invasion and TNM classification. However, rectal cancer resection tended to be performed more frequently in the conventional laparoscopic colectomy group (*p* = 0.091).

**Table 1:** Patient characteristics

Variable	Conventional lap	2-surgeon technique	
	*n* = 429	*n* = 106	*p* value
Mean age (range)	67.9 (27–93)	67.1 (29–80)	0.493
Body mass index (kg/m2)	23.1	22.7	0.21
Gender, Male: n (%)	252 (59 %)	55 (52 %)	0.201
History abdominal surgery n (%)	172 (40 %)	39 (37 %)	0.533
cTNM T factor: n (%)			
0/1	87 (20 %)	16 (15 %)	0.829
2	54 (12 %)	14 (13 %)	
3	247 (58 %)	66 (62 %)	
4	41 (10 %)	10 (10 %)	
cTNM staging: n (%)			
0/I	122 (28 %)	28 (26 %)	0.203
II	105 (25 %)	28 (26 %)	
III	147 (34 %)	44 (42 %)	
IV	55 (13 %)	6 (6 %)	
Procedure			
Right side colectomy	144 (34 %)	46 (43 %)	0.091
Transverse colectomy	18 (4 %)	5 (5 %)	
Left side colectomy	174 (41 %)	42 (40 %)	
Rectal resection	93 (21 %)	13 (12 %)	

The short-term outcomes, including the intraoperative and postoperative findings and pathological results, are shown in Table 2. Reconstruction via extracorporeal FEEA was performed significantly more frequently in the two-surgeon technique group (74 %) than in the conventional laparoscopic colectomy group (57 %). Furthermore, intracorporeal anastomosis using DST was performed significantly more frequently in the conventional laparoscopic colectomy group than in the two-surgeon technique group. A permanent stoma was created for the Hartmann procedure in five patients in the conventional laparoscopic colectomy group. The mean operative time in the two-surgeon technique group (117.9 min) was significantly shorter than that noted in the conventional laparoscopic colectomy group (170 min), as was the time required to perform each of the reconstruction procedures. Intraoperative complications occurred in three patients (3 %) in the two-surgeon technique group, while two patients required the insertion of an additional port due to multiple rectal injuries resulting from transanal insertion of the circular stapler; in these cases, sigmoidectomy was converted to low anterior rectal resection via conventional laparoscopic surgery. Another patient experienced unexpected bleeding, and one patient required conversion to open surgery because of uncontrolled bleeding in the deep pelvic floor. The median postoperative hospital stay was significantly shorter in the two-surgeon technique group (6 days) than in the conventional laparoscopic colectomy group (7 days). There were no major postoperative complications (such as anastomotic leakage, hemorrhage or bowel strangulation) requiring reoperation in the two-surgeon technique group. The mortality rate was zero in both groups. There were no cases of readmission due to the occurrence of late complications, such as ileus or abdominal wall complications, among the cases analyzed in the present study.

**Table 2:** Short-term outcomes, surgical findings, and postoperative findings

Variable	Conventional lap	2-surgeon technique	
	*n* = 429	*n* = 106	*p* value
Reconstruction: n (%)			
FEEA	246 (57 %)	79 (74 %)	0.004
DST	178 (42 %)	26 (25 %)	
stoma	5 (1 %)	1 (1 %)	
Operative time (mean, min)	178.5	117.9	0.0001
operative time under FEEA	170	110.3	0.0001
operative time under DST	187.8	141.4	0.0001
Blood loss (mean; range, g)	19.7 (0–917)	12.3 (0–230)	0.294
Length of umbilical incision (mean, cm)	4.59	4.1	0.0001
Intraoperative complication: n (%)	5 (1 %)	3 (3 %)	
convert to conventional LAP	-	1 (1 %)	
convert to open surgery	5 (1 %)	2 (2 %)	
Postoperative conmlication: n (%)	23 (5 %)	3 (3 %)	0.278
leakage	6 (1.4 %)	1 (1 %)	
hematoma & hemorrhage	5 (1.2 %)	2 (1.8 %)	
paralytic ileus	6 (1.4 %)	0	
Postoperative hospital stay (median; range, day)	7 (5–136)	6 (4–20)	0.0001
Tumor size (mean, cm)	4.0	3.8	0.339
Harvest lymph node (mean, n)	25.2	24.7	0.667
pTNM staging: n (%)			
0/I	120 (28 %)	30 (30 %)	0.066
II	132 (31 %)	31 (29 %)	
III	121 (28 %)	36 (34 %)	
IV	56 (13 %)	7 (7 %)	
R0 resection: n (%)	429 (100 %)	106 (100 %)	-

The pathological findings revealed that the mean tumor diameter and number of harvested lymph nodes did not differ significantly between the two groups. R0 resection with negative histological margins was achieved in all patients. The final TNM stage was similar in both procedures.

## Discussion

Laparoscopic surgery has been used worldwide to treat various diseases since Mouret et al. published their study on laparoscopic cholecystectomy in 1987 [16]. Gradually, laparoscopic surgery has become a standard treatment for colorectal cancer. This approach provides many benefits over open surgery, including lower postoperative pain, better cosmetic outcomes, a decreased incidence of abdominal wall complications, faster return of the bowel function and shorter hospital stay, with similar oncological outcomes. The reduced number of surgeons and ports required for RPS, which is considered to be a more advanced procedure, may make it superior to conventional laparoscopic colectomy.

For the extraction of large tumors or in cases that require extracorporeal anastomosis (such as advanced colorectal cancer), the length of the umbilical incision is determined by the tumor diameter. Therefore, it is logical to base the procedure for laparotomy (such as single-incision laparoscopic colectomy) on the size of the tumor. However, because this surgery involves inserting the required ports into a small opening, interference may occur between the laparoscope and forceps, thereby restricting the surgery and inducing high stress for surgeons who are unfamiliar with the procedure. Excellent surgical technique and teamwork are therefore required to maintain oncological curability. Consequently, this procedure cannot be introduced at all institutions. Of course, expert laparoscopic surgeons can easily understand that RPS, which involves adding one more port to the single-incision laparoscopic colectomy procedure, dramatically improves operability and enables the performance of surgery without introducing unnecessary stress. RPS may also function as a bridge between conventional multiport laparoscopic surgery and single-incision laparoscopic colectomy for training surgeons.

Conventional laparoscopic colon cancer resection is usually performed by three people (one surgeon, a surgeon’s assistant and a laparoscopist). There are no previous studies regarding reducing the number of participating surgeons. Because RPS has, in the past, been safely performed by two surgeons, it should be possible to perform laparoscopic colon cancer resection with two surgeons. In recent years, there has been a growing need to adopt this surgical procedure because the number of colorectal surgeons has decreased due to poor working conditions, while the number of colorectal cancer patients continues to increase each year. At our institution, RPS using the two-surgeon technique was introduced, not for cosmetic reasons, but to develop a safe laparoscopic procedure that requires only two surgeons. This technique must therefore prove to be equal to conventional techniques with regard to safety and the cure rate, and surgeons must not hesitate in adding ports for safety.

The present study demonstrated that it is possible to successfully perform RPS using the two-surgeon technique, regardless of the TNM stage. However, this study is recognized to have major biases, such as the inclusion of patients treated with rectal resection and who required reconstruction using DST in the conventional laparoscopic colectomy group. In addition, the mean operative time in the conventional laparoscopic colectomy group (178 min) was longer than that in the RPS group (118 min), even though the reconstruction times in the two groups were similar. The technique was performed safely and without complications or an increased length of hospital stay. In one case, which involved liver cirrhosis, the operative time was significantly longer; however, this case required three transections at the rectum to repair multiple rectal injuries caused by transanal insertion of the anastomosis instrument. The insertion of surgical instruments up to the rectal stump exposed the shortcomings in operability and problems caused by the limited number of forceps that could be used in the procedure. In the future, replacing the 28 mm head on the anastomosis instrument with a 25 mm head should result in increased stability. These results suggest that RPS using the two-surgeon technique is not well indicated for cases that require DST reconstruction. Conversely, the procedure was found to be well suited to colon cancer cases that require FEEA reconstruction.

Although RPS using the two-surgeon technique is based on conventional laparoscopic colectomy, the “rectal transection first” technique for rectal cancer is a different approach. This point entails delaying the processing of the inferior mesenteric artery in favor of prior mobilization and transection of the rectum. Because the limited operability of the forceps is maximized by maintaining tension on the mesorectum, this technique can be extremely useful for mobilizing the mesorectum on the anal side of the tumor and for achieving transection of the rectum. We experienced no problems with the “rectal transection first” approach. On the other hand, Free Access® is a new attachable platform that affords excellent operability with forceps and can be used for a range of operative procedures, such as small abdominal incisions, based on the tumor diameter. Therefore, it can be used safely without reducing the quality of most colorectal cancer resection procedures, including operations for locally advanced colorectal cancer and DST reconstruction.

The main reason for implementing RPS using the two-surgeon technique was not to reduce patient invasiveness; rather, this decision was driven by our limited number of staff, time constrictions and the increased number of colon cancer patients who require surgery. The procedure described in this report is anticipated to deliver economic benefits, including increased profits due to greater operative throughput. It will also benefit surgeons, through a reduction in the total operative time, which will in turn improve the surgeon’s quality of life by reducing the number of hours spent at work.

## Conclusion

This is the first report to focus on the two-surgeon technique for reduced port laparoscopic colorectal cancer surgery. This technique uses the same instruments, procedures and operative field exposure as conventional laparoscopic colectomy and maintains the same quality, safety and radical curative properties. Furthermore, the procedure applied in this study improved the quality of life of some surgeons by reducing the number of hours they spent at work through a reduction in the number of surgeons involved in the procedure as well as the operative time associated with the two-surgeon technique.
